# The Neuroimmune Interface and Chronic Pain Through the Lens of Production Animals

**DOI:** 10.3389/fnins.2022.887042

**Published:** 2022-05-19

**Authors:** Charlotte H. Johnston, Alexandra L. Whittaker, Samantha H. Franklin, Mark R. Hutchinson

**Affiliations:** ^1^Faculty of Health Sciences, Adelaide Medical School, University of Adelaide, Adelaide, SA, Australia; ^2^School of Animal and Veterinary Sciences, University of Adelaide, Roseworthy, SA, Australia; ^3^Equine Health and Performance Centre, University of Adelaide, Roseworthy, SA, Australia; ^4^Australian Research Council Centre of Excellence for Nanoscale BioPhotonics, University of Adelaide, Adelaide, SA, Australia; ^5^Davies Livestock Research Centre, University of Adelaide, Roseworthy, SA, Australia

**Keywords:** chronic pain, husbandry procedures, livestock, neuroimmune, animal welfare, neuropathic pain

## Abstract

Communication between the central nervous system (CNS) and the immune system has gained much attention for its fundamental role in the development of chronic and pathological pain in humans and rodent models. Following peripheral nerve injury, neuroimmune signaling within the CNS plays an important role in the pathophysiological changes in pain sensitivity that lead to chronic pain. In production animals, routine husbandry procedures such as tail docking and castration, often involve some degree of inflammation and peripheral nerve injury and consequently may lead to chronic pain. Our understanding of chronic pain in animals is limited by the difficulty in measuring this pathological pain state. In light of this, we have reviewed the current understanding of chronic pain in production animals. We discuss our ability to measure pain and the implications this has on animal welfare and production outcomes. Further research into the neuroimmune interface in production animals will improve our fundamental understanding of chronic pain and better inform human clinical pain management and animal husbandry practices and interventions.

## Introduction

Pain is defined by the International Association for the Study of Pain (IASP) as “an unpleasant sensory and emotional experience associated with, or resembling that associated with, actual or potential tissue damage. “ The definition has recently been modified to acknowledge the experience of pain in a broader population, including those unable to verbally describe their experience (Raja et al., 2020). This marks a philosophical, scientific, and societal shift in the understanding of pain in infants and animals alike. There is growing evidence demonstrating the emotional and cognitive elements of the subjective pain experience in non-verbal humans and animals (Neave et al., 2013; Adcock and Tucker, 2020a) and the potential consequences of misinterpreting the sometimes subtle, absent, or overlooked expression of pain (Taddio et al., 1997; Viñuela-Fernández et al., 2007; Fitzgerald and McKelvey, 2016).

Acute pain is an adaptive process that protects the organism from further injury and promotes healing through behavioral changes. Nociception is the neural process of surveying and encoding noxious signals from the external and internal environment eliciting reflexes for rapid response to noxious stimuli and transmission to higher centers for more advanced interrogation and response to the signal. Nociception can alter behaviors or result in no action, and it does not always lead to the unpleasant experience of pain. Nociception is vitally important to normal function as it protects the organism against injury (Baliki and Apkarian, 2015). Nociceptive signals are processed by multiple regions of the brain in the context of the psychological and environmental state of the individual resulting in the complex multidimensional experience of pain. Here, nociceptive pain refers to the pain experienced following activation of nociceptors in response to actual or potential tissue damage.

Acute pain that persists past the expected resolution is maladaptive and is referred to as chronic pain or persistent pain. Changes in the peripheral and central nervous system (CNS) as well as bi-directional communication between the immune system and the CNS result in the development of chronic pain (von Hehn et al., 2012; Grace et al., 2014). Chronic pain serves no physiological function and is detrimental to the wellbeing of the individual (Cohen et al., 2021). In humans, chronic pain is defined by IASP as pain persisting more than three months and causing emotional distress and/or functional disability (Treede et al., 2015). Chronic pain is a significant and growing problem in humans, that affects between 11 and 40% of the population (Cohen et al., 2021; Gregus et al., 2021; Herzberg and Bustamante, 2021). Patients with this condition often suffer from mental illness and cognitive deficits including depression, anxiety, catastrophizing, sleep disturbance, and memory impairment (von Hehn et al., 2012; Navratilova et al., 2015). Significant losses in work productivity and reduced capacity to carry out daily tasks such as childcare and housekeeping have also been associated with chronic pain (Kronborg et al., 2009). The economic burden of chronic pain in the United States alone is estimated at $560–635 billion in medical bills and lost productivity annually (Treede et al., 2015; Gregus et al., 2021). Addressing the public health concern of chronic pain is hampered by the difficulty diagnosing and treating the condition. This is due to the marked variability of clinical disease between individuals and the contribution of external factors such as a patient’s social environment and culture to the experience of pain. Consideration of the biological, psychological, and social influences on a patient’s experience of chronic pain using the biopsychosocial model, has greatly improved the understanding, prevention, diagnosis, and treatment of pain (Bevers et al., 2016).

In production animals, such as sheep, cattle, and pigs, the term chronic pain is used more broadly, often to describe pain persisting past the point of tissue healing or past the acute phase (Kent et al., 1999; McCracken et al., 2010; McLennan, 2018; Williams, 2019; Herzberg et al., 2020a; Adcock, 2021), with less focus on a specified time point. A well-recognized example of chronic pain in livestock is lameness caused by various pathogens and environmental factors including *Dichelobacter nodosus* causing footrot in sheep and osteoarthritis of infectious, traumatic, or degenerative origin (Herzberg et al., 2020a). A more unique aspect of the life of a farm animal compared to humans, is the necessary husbandry practices required to manage animals in a production system. This often involves the use of surgical techniques across the whole population to alter their phenotype and reduce the risk of disease or injury. Examples of this include dehorning to prevent injury to other animals and stock people, castration, and tail docking and mulesing to reduce the build-up of fecal material on the tail and breech leading to myiasis (flystrike). Tail docking involves amputating the end of the tail at the third palpable intercoccygeal joint and mulesing, which may be performed in addition to tail docking, is the removal of wrinkled wool-bearing skin around the breech and the edges of the tail. Often these procedures are performed with minimal or no analgesia or anesthesia and pain lasting longer than 12–24 h, which is expected as a result of surgical tissue damage, has largely been overlooked (Viñuela-Fernández et al., 2007). Several of the detrimental effects of chronic pain in humans, including anxiety, depression and cognitive impairments could also occur in animals, and would negatively affect productivity and welfare. Research into the long-lasting effects of painful husbandry procedures in livestock is limited (Viñuela-Fernández et al., 2007; Herzberg et al., 2020b; Small et al., 2021). However, growing interest in improving the welfare of animals is driving further investigation of the long-term consequences of various husbandry practices and chronic disease in livestock (Troncoso et al., 2018; Larrondo et al., 2019; Sandercock et al., 2019; Herzberg et al., 2020a).

Industry relies on support from the community, government, and stakeholders to operate and generate profit. Social license to operate (SLO) refers to the informal approval given by the community to an industry to enable ongoing activity and this can change depending on prevailing values (Hampton et al., 2020). Maintaining SLO requires consistent and proactive engagement with community, promotion of open and honest communication, as well as education of those involved in the industry and not only consumers but also the wider community. Interaction with the scientific community is vital to demonstrate commitment to identifying concerns and responding appropriately (Hampton et al., 2020). Animal welfare issues are one of the main contributors to deterioration of SLO. For example, mulesing and live animal export have received considerable attention following media-led exposés, leading to widespread loss of confidence in the related industries and boycotts on products. Pre-empting threats to SLO is important for industry to avoid reactionary measures and adverse publicity, which cause significant economic losses and require substantial investment of time and resources to regain consumer confidence (Hampton et al., 2020).

In addition to the social license required for ongoing animal production, government and stakeholder support are crucial. Legislative mandates direct minimum standards. Changing these standards requires robust evidence and overwhelming support from multiple groups. Animal welfare standards differ markedly between countries and even within countries. Welfare standards still differ considerably between countries, and this can affect trade (Peters, 2016). For example, exposé type journalism revealed mulesing practices in Australia, leading to global outrage and boycotting of Australian wool products in some countries (Hampton et al., 2020). These sorts of reactionary approaches often stem from disengaged industries and misinformed responses from groups and individuals outside of the industry – abrupt bans and boycotts ultimately harm global relations, industry, communities, and animals (Windsor, 2021). Uncovering potential welfare concerns using a scientific approach followed by open communication and engagement between interested parties often leads to sustainable improvements or alternatives to practice that benefit animals, the industry, and consumers (Windsor, 2021).

Identifying and addressing animal welfare concerns, such as acute and chronic pain, requires reliable objective measurements for creating effective solutions and monitoring progress (Windsor, 2021). Objective measurements of pain are difficult as pain itself is a subjective experience and animals are unable to describe that experience (Grace et al., 2010). When it comes to measuring the long term impacts of disease and various practices involved in managing large groups of animals in a productive system the current methods available to us rely largely on subjective empirical data and there is still a lot that is unknown (Windsor, 2021), particularly when it comes to persisting pain or chronic pain conditions (Kleinhenz et al., 2021). Objective and reliable measures of pain would present exciting opportunities to assess pain and address it where necessary. These measures would contribute significantly to developing new treatments and techniques to mitigate pain and improve our understanding of the experience of pain in animals (Hutchinson and Terry, 2019). Not only would objective measures of pain greatly benefit animals, humans may also reap the rewards as livestock present a larger model of human disease (Reddy et al., 2018; Herzberg and Bustamante, 2021).

Advances in our understanding of pain neurobiology have uncovered opportunities to objectively measure pain. Quantifying the neuroimmune synapse has proven successful at identifying states of chronic pain in both rodents and humans (Grace et al., 2010, 2011; Kwok et al., 2012), and it may also be true in livestock. The neuroimmune interface describes the communication between immunocompetent cells in the CNS, the peripheral immune system, and the nervous system. The immune system is vital for regulating normal function of the nervous system and is also the first responder to damage within this system. Bi-directional communication between these systems contributes to biological coherence, explaining the influence of the immune system on behavior and vice-versa (Hutchinson and Terry, 2019).

This hypothesis generating review seeks to give an overview of the current state of play in terms of pain assessment in livestock, highlighting the gaps in knowledge surrounding the pathophysiology of pain states in production animals, and the potential that future advances in livestock neuroscience can improve animal welfare and human clinical pain interventions and diagnosis.

## Measuring Pain in Livestock

**FIGURE 1:**
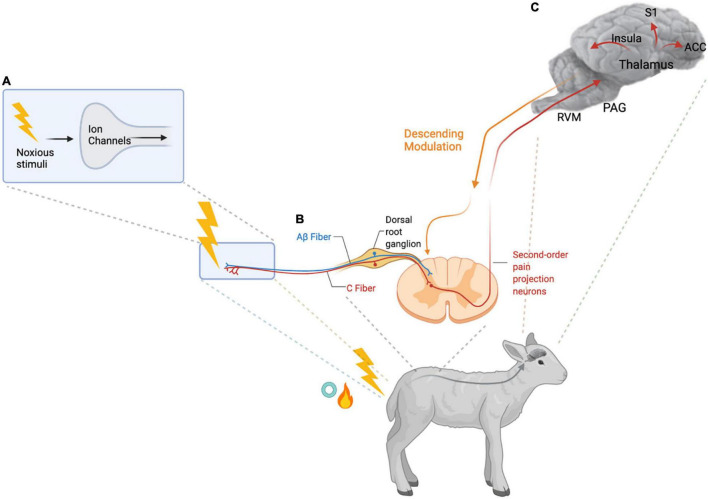
Nociception and Pain Processing. (A) High threshold nociceptive sensory neurons (first-order primary afferent neurons) are activated by noxious stimuli such as the thermal, mechanical, and inflammatory stimuli of hot knife or rubber ring tail docking in lambs. Activation of the ion channels on the peripheral terminals of these nociceptors results in transduction of the signal. (B) The ionic signal is then transmitted along the nociceptive C- and Aβ fibers *via* the dorsal root ganglion to the spinal dorsal horn. In the spinal dorsal horn, the central terminals of the first-order primary afferent neurons synapse with inhibitory interneurons and second-order pain projection neurons which travel up to the brain. (C) Information about the duration and intensity of the noxious stimuli is transmitted *via* the brainstem and thalamus to cortical and subcortical regions of the brain including the anterior cingulate cortex (ACC), the insular cortex, and the somatosensory cortex (S1) to generate the sensory and emotional dimensions of pain. Several brainstem regions including the rostral ventromedial medulla (RVM) and the periaqueductal gray (PAG), contribute to descending modulation of the pain signal (von Hehn et al., 2012; Grace et al., 2014). Created with BioRender.com.

Pain is an adaptive mechanism to protect an individual from harm and promote healing. When an individual is exposed to a noxious stimulus, ion channels on the cell membrane of high threshold sensory neurons in the skin or viscera are activated. An ionic signal is then conducted along these first order primary afferent neurons to central synapses in the dorsal spinal horn *via* dorsal root ganglia (DRG). The nociceptive signal is transmitted through second order nociceptive projection neurons to supra-spinal sites, which project to cortical and subcortical regions *via* third order neurons. Inhibition of nociceptive signals can occur at the level of the spinal cord through activation of GABAergic and glycinergic inhibitory interneurons or through activation of descending serotonergic and noradrenergic projections to the spinal cord (Figure 1). In the brain the signal is transformed in the context of the emotional state of the individual resulting in the multidimensional pain experience (Grace et al., 2014; Frias and Merighi, 2016; Porreca and Navratilova, 2017). The brain regions involved in processing nociceptive signals include the anterior cingulate cortex, the insula, and the thalamus. These areas have connections to the mesolimbic system, composed of the ventral tegmental area and the nucleus accumbens, which are involved in learning and reward (Baliki et al., 2010; Porreca and Navratilova, 2017). The rostral ventromedial medulla modulates descending nociceptive signals. The processing of nociceptive signals ultimately alters behavior in ways that should benefit the individual but can become harmful in pathological states such as chronic pain. Chronic pain can be linked to existing or prior tissue injury, in which case it is categorized as nociceptive or neuropathic if nervous tissue is injured or diseased. Sensitization of the normal nociceptive pathways where an obvious tissue injury is not apparent is referred to as nociplastic pain (Treede et al., 2015). Inflammatory pain is also commonly involved in chronic pain conditions, this pain is caused by release of inflammatory mediators after tissue injury. Peripheral and central sensitization are features of chronic pain leading to clinical manifestations of hyperalgesia and allodynia (Table 1) (von Hehn et al., 2012).

**TABLE 1:** Pain definitions (Treede, 2006).

Peripheral sensitization	Reduction in threshold and increased excitability at the peripheral terminals of nociceptive neurons. Commonly associated with inflammatory pain.
Central sensitization	Increased responsiveness of nociceptive neurons in the central nervous system to normal or subthreshold stimuli
Primary hyperalgesia	Increased sensitivity to a painful stimulus at the site of tissue damage
Secondary hyperalgesia	Increased sensitivity to a painful stimulus distant from the site of tissue damage, indicative of central sensitization
Allodynia	Pain due to a non-painful stimulus

Assessing pain in animals, is notoriously difficult and frequently under recognized or overlooked. (Grace et al., 2010; Hutchinson and Terry, 2019; Williams, 2019; Adcock, 2021; Steagall et al., 2021; Tschoner, 2021). This difficulty is often attributed to livestock being prey species which hide behavioral evidence of pain to avoid predation (Williams, 2019; Cohen and Beths, 2020; Steagall et al., 2021). However, Carbone (2020) argues that the relationship between the observer and the animal, be it prey, predator or both, is the major limiting factor for accurate diagnosis of pain. When comparing pain assessment in companion animals, such as cats and dogs, to livestock the main difference is the amount of time an owner spends in close association with their pet/s as opposed to a farmer often caring for hundreds to thousands of animals simultaneously. For example, a cat that would normally jump onto a bench gradually stops this behavior over a matter of weeks to months with no other overt changes in behavior. This reduced activity can be associated with the progression of osteoarthritis causing pain induced by jumping up and down from a height (Monteiro and Steagall, 2019). These subtle changes would typically go unnoticed without appreciation for an individual animal’s normal routine and behavior. Diagnosis of this pain based solely on clinical exam and observation in the exam room would be difficult due to suppression of signs of chronic pain in the clinical setting (Monteiro and Steagall, 2019). Historically, this dampened expression of pain is likely to have resulted in an underestimation of an animal’s ability to experience pain in the same rich multi-dimensional way as humans and thus resulted in the belief that animals were insensitive to pain (Weary et al., 2006). Accurate assessment of pain in animals requires substantial time commitments to create trusting relationships with individual animals and an in-depth knowledge of their normal and abnormal behaviors (Carbone, 2020). This approach is economically and practically unfeasible in most livestock production systems and research settings where large groups of animals are typically housed in groups, observed from a distance, and handled in relatively stressful time-sensitive environments. Marked perturbations from normal behavior specific to the species or based on the observers’ experience are usually readily detected but the subtle changes and differences in individual pain experience typically associated with chronic pain may be missed without focused individual assessment (Monteiro and Steagall, 2019). We know chronic pain negatively affects human wellbeing (Cohen et al., 2021) and animal welfare (Monteiro and Steagall, 2019) and that this form of pain can be incredibly difficult to diagnose and manage (Kronborg et al., 2009; Monteiro and Steagall, 2019). Given the difficultly identifying pain in animals, particularly in livestock, it is possible that an unknown proportion of these animals will be affected by maladaptive pain that serves no purpose and only diminishes welfare and productivity (Viñuela-Fernández et al., 2007; Williams, 2019). Improving our understanding of this form of pain and developing diagnostic and management tools to mitigate this pain will greatly benefit animals. Humans also stand to benefit from pain research using animal models of human chronic pain in larger species with more complex environments closer to the experience of humans as opposed to current standards of preclinical research which serve an important role of highly controlled experiments but often fail to translate to clinical trials (Herzberg and Bustamante, 2021).

The pain experience is unique to an individual and can be influenced by external factors. In humans, behavioral response and the reported experience of pain to similar noxious stimuli is influenced by various factors including social context (Koban and Wager, 2016), culture, education, stoicism, personality, sex, and environmental circumstances (Linton and Shaw, 2011). Similarly, it has been shown that expression of pain in animals differs depending on a number of influences, including temperament (Ijichi et al., 2014), affective state (Lecorps et al., 2020) and social interactions (Guesgen et al., 2014). This variability among individuals can lead to difficulty interpreting and comparing the experience of pain from a specific noxious insult across a group of animals.

In humans, the diagnosis of acute pain is often performed through self-report of symptoms and use of the visual analog scale and numeric rating scale (Bendinger and Plunkett, 2016). Chronic pain diagnosis presents a greater challenge, requiring a multifaceted approach to quantify sensory, cognitive and psychological components, and even with specialized tools and a cooperative human patient it can be difficult to tease apart the complex syndromes and various associated comorbidities (Bendinger and Plunkett, 2016; Carnago et al., 2021). These methods of diagnosing acute and chronic pain are often inaccurate or not possible in animals and in humans that are mentally incapacitated, preverbal neonates, or unable to precisely communicate their experience. In animals, diagnosing pain has traditionally been done using behavioral observation, and physiological and neuroendocrine changes. These measures can be insensitive, often discount the long-term effects of pain, and overlook the cognitive and psychological components of pain (Weary et al., 2006; Steagall et al., 2021; Tschoner, 2021). More recently, the psychological and affective state elements of pain in livestock have been studied to assess the changes in cognition, motivation and decision-making that are known to occur due to higher-order processing of nociceptive signals (Neave et al., 2013; Sneddon et al., 2014; Adcock and Tucker, 2020a; Lecorps et al., 2020; Kleinhenz et al., 2021).

### Behavioral Measurements of Pain

Observation and quantification of behavior is commonly used for measuring acute and persistent pain in livestock. Behavioral observation is a readily accessible and useful form of measurement and while time consuming, it requires minimal intervention and equipment. Typically, behaviors indicating pain are described broadly and then counted or scored across a group of individuals using an ethogram. The ethogram should be tailored to the noxious stimulus or treatment being studied as behaviors differ depending on the type and duration of pain experienced. Pain behaviors are adaptive responses aimed at escaping, avoiding or minimizing pain; typically these behaviors are tailored to the location, type, and severity of the lesion (Molony and Kent, 1997). For example, the type of behavior employed in response to acute ischemic pain induced by rubber ring castration commonly involves increased active pain behaviors such as stamping, rolling and greater time spent lying abnormally; whereas incisional pain caused by mulesing results in more immobile standing postures (Grant, 2004; Paull et al., 2007; Lomax et al., 2010). Consequently, comparing different types of procedures or painful conditions using behavioral observation alone can be difficult and misleading (Small et al., 2021). Expression of pain behaviors can also be suppressed in the presence of a greater threat or fear (Grandin and Deesing, 2003; Rea et al., 2013). For example, lambs that have been castrated and are displaying signs of severe pain will stand up and run as though pain-free when the rest of the flock is moved. Behavioral response to acute pain also changes over time and typically becomes more subtle or even absent with milder forms of pain. In the case of maladaptive or chronic pain, these behaviors can be paroxysmal and heterogenous (von Hehn et al., 2012) and so may be missed if animals are observed intermittently. The development and onset of chronic pain also varies between individuals and lesion types (von Hehn et al., 2012). In an ovine model of neuropathic pain, when the common peroneal nerve was either constricted or severed, mechanical hypersensitivity only developed 20-30 days after the nerve injury (Reddy et al., 2018). In lambs castrated and tail docked using rubber rings and no form of anesthesia or analgesia there was a significant increase in summated total active behaviors compared with handled controls at 10-41 days after treatment (Kent et al., 2000). Active behaviors included tail wagging, foot stamping and kicking, scratching hindquarters, and easing quarters. These behaviors were measured in eight 3-h periods across the 31 day period starting 10 days after treatment (Kent et al., 2000). Detecting these subtle behavioral changes is time consuming and can be difficult to interpret without additional physiological and behavioral data (Petherick et al., 2014).

Facial expression and ear position are receiving more attention as sensitive indicators of pain in livestock (Guesgen et al., 2016b; Viscardi et al., 2017; McLennan, 2018). Facial expressions related to pain may reflect the negative emotional component of pain (Langford et al., 2010; Cohen and Beths, 2020). This was demonstrated in mice where lesioning the rostral anterior insula, an area of the brain associated with the affective component of pain in humans, resulted in attenuation of grimace score without influencing abdominal writhing following intraperitoneal injection of 0.9% acetic acid (Langford et al., 2010). Grimace scales have been developed for several species to score changes in specific facial action units to rate overall pain in an individual (Guesgen et al., 2016b; Viscardi et al., 2017; McLennan, 2018). The facial action units commonly used to indicate pain are orbital tightening, cheek flattening or bulging, nostril and chin tension, and backward ear position (Cohen and Beths, 2020). These changes in facial action units are generally conserved across species including horses (Dalla Costa et al., 2014), sheep (Guesgen et al., 2016b; McLennan et al., 2016), cattle (Gleerup et al., 2015), and pigs (Viscardi et al., 2017). Changes in facial expression occur rapidly and it is likely that the changes are involuntary, as is seen in humans, where the facial expression of pain is difficult to hide or consciously influence (McLennan, 2018). Grimace scales have been shown to be valid and have good inter-observer reliability (Dalla Costa et al., 2014; Häger et al., 2017). The spontaneity, versatility, and sensitivity of this tool lends well to automation, allowing improved surveillance of health and disease to advance animal welfare. Additionally, remote recording and computer algorithms of changes in facial action units removes the influence of the observer on the animal and the potential for observer bias (McLennan and Mahmoud, 2019).

There are some notable limitations to grimace scales; the presence of the observer and the environment can influence the attention and facial expression of the animal and changes may be unrelated to pain. In one study, lambs observing a related lamb in pain spent more time with their ears backward (Guesgen, 2015), which has been shown to be an indicator of negative emotional state associated with pain (Guesgen et al., 2016a). Aggression and sedation have also been known to cause false positives when using the grimace scale (Cohen and Beths, 2020). Thus, it is recommended to monitor the animal for a short period of time to score the most relevant expressions and be aware of species differences and the context of the testing scenario. Facial grimace scales are rapid and useful indicators of pain that allow timely intervention and monitoring of response to that intervention. However, observer bias and environmental influence can make results difficult to interpret or false when facial expression is used as a sole measurement tool.

Telemetry and geolocation devices can be useful for identifying changes in behavior associated with reduced welfare and pain (Petherick et al., 2014; Small et al., 2021; Tschoner, 2021). Significantly reduced activity has been demonstrated in an osteoarthritis model in sheep (Newell et al., 2018), and following castration in calves and bulls (Currah et al., 2009; Petherick et al., 2014). General activity levels change in response to other factors, such as the weather, estrus, and stress (Tschoner, 2021). As with other behavioral measures, objective data from these remote tracking devices is useful and sensitive but needs to be interpreted with caution.

### Nociceptive Threshold Measurements to Quantify Pain

Quantitative sensory testing (QST) is commonly used in pain research and involves testing an animal’s response to a ramped mechanical, thermal, or electrical stimulus and recording the intensity of the stimulus applied (Johnson, 2016; Taylor, 2020). Quantitative sensory testing is useful for assessing the development of hyperalgesia and allodynia in response to a noxious stimulus, and it is also used to investigate resolution or reduction in nociceptive thresholds over time or with interventions, such as analgesic agents. Quantitative sensory testing does not always capture the cognitive component of pain but reliably measures changes in nociception. Quantitative sensory testing has been used in sheep (Lomax et al., 2008, 2010, 2013), dairy cows (Eicher et al., 2006; Adcock and Tucker, 2018; Troncoso et al., 2018), and pigs (Sandercock et al., 2011; Di Giminiani et al., 2017a) to identify hyperalgesia following a variety of husbandry procedures including mulesing, castration and tail docking. Quantitative sensory testing has also demonstrated prolonged hyperalgesia in chronic illness, such as lameness (Ley et al., 1995; Whay et al., 1998).

Ideally, the research environment should be strictly controlled to obtain the most reliable and repeatable results from QST. This involves minimizing distractions such as noise and unfamiliar smells, animals or people, allowing subjects time to acclimatize to their environment, and maintaining a regular ambient temperature (Johnson, 2016). Distraction or external threat, particularly in prey animals, can alter response to stimuli; generally leading to reduced focal pain sensitivity which equates to increased nociceptive thresholds (Johnson, 2016; Taylor, 2020). When testing livestock, it is often necessary to restrain them in unfamiliar environments and it is usually not economically feasible or ethical to manage them in stringently controlled research environments, as is possible with rodents. Due to the practicalities of studying husbandry procedures in livestock research, animals are frequently tested in yards which can be noisy with extremes in temperature, and young animals may be separated from their mother and familiar animals to be restrained in unfamiliar positions, such as a marking cradle. The animals are usually retested within hours to days of the procedure in the same restraint and may be vaccinated at the time of treatment. Vaccines trigger an innate and subsequent adaptive immune response which can reduce nociceptive thresholds (Hervé et al., 2019) and therefore may affect QST testing in the days following a painful insult. Despite this, numerous studies using QST in commercially managed livestock have been able to reliably identify pain following painful procedures (Adcock and Tucker, 2018) and responses to analgesics (Lomax et al., 2010, 2013; Gottardo et al., 2016; Sheil and Polkinghorne, 2020; Stock et al., 2021).

### Physiological Measurements to Quantify Pain

Response to stress and pain can be modulated by the sympathetic-adrenomedullary system and the hypothalamo-pituitary-adrenocortical (HPA) system. Changes in the sympathetic system can be measured through autonomic responses including heart rate, heart rate variability (von Borell et al., 2007; Stubsjøen et al., 2009), blood pressure (Peers et al., 2002), respiratory rate and ocular temperature (Harris et al., 2021; Stock et al., 2021). These measures are more sensitive for mild pain compared to behavioral assessment and HPA axis changes (Peers et al., 2002), particularly in prey species (Weary et al., 2006; Sneddon et al., 2014). However, they are not specific to pain and can change dramatically with stress (Weary et al., 2006) or other forms of disease such as septic shock or blood loss (Johnson, 2016). Therefore, interpreting changes in autonomic responses should be done with consideration of the environmental context, treatment, variations in experience of handling, and any underlying conditions that may alter response.

The HPA system responds to stress and pain by release of adrenocorticotrophic hormone (ACTH) from the pituitary gland, which then stimulates release of cortisol from the adrenal gland. Cortisol has been used extensively to assess pain and stress in livestock (Ley et al., 1994; Kent et al., 1995, 1998; Dinniss et al., 2000; Peers et al., 2002; Becker et al., 2012). In studies of the pain associated with husbandry procedures including mulesing, castration and tail docking, plasma cortisol typically rises acutely following the procedure and then returns to normal levels by 2-4 h (Mellor and Stafford, 2000; Molony et al., 2002; Becker et al., 2012). Elevation in plasma cortisol does not always correlate well with pain related behaviors and differences in results may be due to method or other contributing factors, such as stress, rather than the level of pain experienced (Mellor and Stafford, 2000). In cases of chronic disease leading to chronic pain, plasma cortisol levels can be conflicting, in one study cortisol levels were significantly higher in sheep with footrot compared to healthy controls, but did not correlate with the severity of disease (Ley et al., 1994). Whereas, in a similar study by the same group cortisol levels were lower in severely lame sheep compared to controls (Ley et al., 1991). Depression of plasma cortisol levels has also been associated with chronically poor welfare states in horses including those diagnosed with back pain (Pawluski et al., 2017). Cortisol is an important hormone with numerous endocrine actions and diurnal patterns that maintain homeostasis in times of stress. Cortisol levels can be used as a measure of stress associated with painful interventions but should be interpreted in light of other measures of pain and it may be difficult to use as a reliable measure of chronic pain or stress.

There is evidence that measuring cortical electrical activity, *via* an electroencephalogram (EEG), allows objective quantification of the cognitive perception of pain in livestock (Jongman et al., 2000; Gibson et al., 2007; Johnson et al., 2009). Frequency alterations in EEG signal are interpreted using spectral analysis. The variables F50 and Ptot are correlated with noxious stimulation (Gibson et al., 2007) and F95 is correlated with depth of anesthesia. F50 is the median frequency, F95 is the spectral edge frequency, and Ptot is the total area under the power spectrum curve. In lightly anesthetized lambs and calves, significant increases in F50 and F95 and significant decrease in Ptot occurred after noxious stimuli – surgical castration in lambs (Harris et al., 2020) and dehorning in calves (Gibson et al., 2007). In conscious lambs that were surgically castrated EEG was able to differentiate the effect of castration and the use of the local anesthetic, lidocaine (Harris et al., 2020). However, in a later study of surgical castration in conscious lambs on farm there was no significant effect of treatment on EEG outcomes between surgical castration without analgesia, surgical castration with lidocaine and meloxicam, and sham castration (Harris et al., 2021). The difference in findings were attributed to the effect of the controlled research environment used for the first trial and the more stressful farm environment in the second trial which may have made the results difficult to interpret. EEG may be useful for analyzing pain perception but currently its use is confined to a research environment (Small et al., 2021).

### Cognitive Measures to Quantify Pain

Measuring the aversiveness of pain in livestock is receiving more attention as it may be a better indicator of the affective component of pain (Ede et al., 2019; Adcock and Tucker, 2020a). It is also a recognition that the emotional state of an animal impacts their welfare and reflects animal welfare science dogma moving toward ensuring a life worth living for livestock (Mellor, 2016). Pain motivates changes in behavior in the same way as hunger and thirst, driving decision making and learning important for survival (Porreca and Navratilova, 2017). Tests developed in rodents aimed at assessing the motivational and emotional components of pain include the conditioned place preference learning paradigm (CPP). The premise being, animals with ongoing pain will chose to spend more time in a place associated with relief of that pain (Porreca and Navratilova, 2017; Adcock and Tucker, 2020a).

Conditioned place preference tests in calves following disbudding demonstrated ongoing pain three weeks after treatment (Adcock and Tucker, 2020a). Conditioned place aversion in disbudded calves differed between analgesic agents of the same class based on duration of action. The non-steroidal anti-inflammatory drugs (NSAIDs) meloxicam and ketoprofen were used in this study. These calves, tested 2 days after disbudding and conditioning, avoided the area associated with the shorter acting analgesic (ketoprofen) that would have been ineffective at preventing pain for the whole conditioning period (Ede et al., 2019). Given the findings of Adcock and Tucker (2020a) that pain persists for at least three weeks after disbudding, it is possible that aversion may develop with the longer acting analgesic (meloxicam) if the conditioning period was extended. Both studies indicate that the long-term welfare of these calves is negatively impacted by routine husbandry procedures and there should be greater research on the relief of persistent pain as well as acute pain (Ede et al., 2019; Lecorps et al., 2020).

The affective state of an animal may alter its experience of pain (Lecorps et al., 2020) and conversely, pain can alter affective state. Chronic pain has been shown to negatively influence affective state and reward circuits in rodents and humans (Porreca and Navratilova, 2017). Judgment bias tests (JBT) are frequently used to assess affective state in animals by conditioning them to associate two reference cues with a reward, stressor, lesser reward, or no reward and then recording their response to intermediate ambiguous stimuli. For example, Neave et al. (2013) used a white or red screen as cues for a JBT in dairy calves, and adjusted the saturation level of red to 25%, 50%, and 75% to act as ambiguous cues. Individuals in a positive affective state tend to have an enhanced expectation of a good outcome and will view ambiguous stimuli positively, whereas those in a negative affective state expect a poor outcome and so tend to interpret ambiguous stimuli negatively (Baciadonna and McElligott, 2015). Studies in dairy calves have looked at these cognitive tests to evaluate the effects of disbudding on affective state. Neave et al. (2013) found that calves developed a negative bias for at least 22 h after disbudding. Lecorps et al. (2020) looked at innate affective state in dairy calves as a measure of personality, finding that more pessimistic calves experienced greater pain-induced anhedonia (reduced or absent ability to derive pleasure from reward) than their optimistic counterparts. They also showed that pain-induced anhedonia, in this case the reduction in consumption of a rewarding sweet solution, persisted for at least 5 days after disbudding. In both studies only short acting anesthesia (xylazine) and a local anesthetic (lidocaine) were used for the disbudding procedure.

### Production Measures to Quantify Pain

Production measures portray the financial and economic costs of pain on a system. This has traditionally been one of the major drivers of change for stakeholders (Steagall et al., 2021). Change in body weight is commonly used as a marker of altered productivity. When animals are in persistent negative affective states and/or pain their feed intake usually decreases leading to a loss in body weight. The evidence for weight loss or reduced growth following husbandry procedures performed without analgesia is equivocal (Small et al., 2020; Stock et al., 2021). Bates et al. (2015) demonstrated improved milk consumption and average daily weight gain over 15 days in dairy calves that were disbudded by farmers and given NSAIDs as analgesia opposed to calves that were not given NSAIDs. Whereas Stock et al. (2021) found no significant difference in average daily weight gain over eight days in dairy calves after disbudding when NSAIDs were used. Piglets castrated and treated with lidocaine and adrenaline 10 min before the procedure had significantly greater average daily weight gain compared to piglets castrated without anesthesia, but this was only evident at 102 days of age (Telles et al., 2016). The authors of this study speculated that this improvement in weight gain was due to reduced peripheral and central sensitization in piglets treated with anesthetic (Telles et al., 2016). A number of other studies comparing piglets castrated with or without lidocaine have not found a difference in average daily weight gain, although these studies only measured weight gain at most up to 7 weeks of age (Kluivers-Poodt et al., 2012, 2013; Abendschön et al., 2020). In adult livestock weight loss tends to occur in chronically painful conditions such as lameness (Nieuwhof et al., 2008; Whay and Shearer, 2017). Compensatory weight gain can occur with successful treatment of the underlying cause of lameness (Nieuwhof et al., 2008). However, the costs of treatment and management changes in addition to a period of reduced feed efficiency and poor welfare likely limit overall economic benefit and highlight the importance of preventative strategies and early diagnosis.

Other production measures, such as survival rate are strong motivators to introduce pain mitigation strategies in livestock practice. The use of NSAIDs after castration and tail docking in lambs significantly improved lamb survival between marking and weaning in one study (Small et al., 2020). Other studies investigating lamb mortality following marking with or without analgesia have failed to find a significant difference in survival. However, these studies were only 28 days in duration (Lomax et al., 2008, 2010) compared to 75–99 days (Small et al., 2020). Neonatal morbidity and mortality causes significant production losses and is detrimental to animal welfare (Mellor and Stafford, 2004). This highlights the importance of further research on analgesia in livestock husbandry procedures and the potential long-term effects on animal welfare and productivity.

Comparatively, human production measures could be considered to include work productivity, use of aids and services such as childcare and housekeeping, and health care costs reflecting changes to everyday function. Like production measures in animals, these parameters can be impacted by a wide range of influences and should be interpreted carefully. Unlike animals, humans are generally able to provide clear reasoning for their actions and so it is easier to relate cause and effect. Chronic pain is associated with significantly reduced productivity at work and increased health care costs and use of support services (Kronborg et al., 2009) as well as significant decline in wellbeing (Porreca and Navratilova, 2017). These are generally long-term effects seen in patients suffering with chronic pain for months to years (Kronborg et al., 2009; Cohen et al., 2021).

The array of pain assessment tools for livestock is considerable and clearly demonstrates the complex and diverse nature of pain in these species. There is a very limited understanding of chronic pain states in livestock. This is partly due to the difficulty in diagnosis and measurement, with most measurement tools focused on acute pain and are insensitive to mild-moderate pain. Pre-clinical chronic pain research has predominantly utilized small animal models. However, there is growing interest in production animal species focusing on developing larger animal models of neuropathic pain for translational studies (Wilkes et al., 2012; Reddy et al., 2018; Chakrabarti et al., 2021). Additionally, a greater understanding of the neuroimmune contribution to chronic pain in rodents and humans is providing insight into this condition and creating new measurement techniques and treatment opportunities that may also be applicable in livestock.

## The Neuroimmune Interface and the Transition From Acute to Chronic Pain

The contribution of the immune system to the functioning of the central nervous system (CNS), known as central immune signaling, is vital and has become an area of growing interest, particularly in relation to the understanding of pain and chronic pain (Grace et al., 2014; Hutchinson and Terry, 2019). Both preclinical rodent studies and clinical human studies have provided strong evidence that maladaptive chronic pain is driven by centrally mediated neuroimmune mechanisms (Grace et al., 2021). The following section gives an overview of neuroimmune engagement in pain propagation and maintenance [for comprehensive review, see Milligan and Watkins (2009), Grace et al. (2011, 2014), Ji et al. (2016), Inoue and Tsuda (2018), and Baral et al. (2019)].

Glial cells in the central nervous system were traditionally seen as structural support cells and little was known about them despite making up greater than 70% of the total cells within the CNS (Milligan and Watkins, 2009; Nicotra et al., 2012). When the peripheral cytokine, interleukin-1β (IL-1β), was implicated in sickness behaviors and found to act in the CNS, glia received more attention as a potential source of centrally acting proinflammatory mediators (Grace et al., 2014). This led to a burgeoning of research investigating the bi-directional communication between immunocompetent cells and neuronal cells, replacing the previously held dogma that the brain was immune privileged. Immunocompetent cells within the CNS include microglia, astrocytes, oligodendrocytes, endothelial cells, perivascular macrophages, and infiltrating T cells. These cells monitor and respond to any alterations within the CNS environment. A rapid and efficient immune response is important for resolution of neuronal insult or injury; however, when there is an aberration in normal immune signaling, pathological pain can develop.

Following tissue injury, immune cells release inflammatory mediators, such as IL-1β and tumor necrosis factor (TNF), which are detected by molecular receptors at the peripheral terminals of afferent nociceptors. This results in excitatory signals being conducted along the nociceptor axons to the dorsal horn of the spinal cord *via* the DRG, leading to increased neuroexcitability driving peripheral sensitization (Baral et al., 2019). High threshold activation of these first-order neurons or peripheral nerve injury stimulates release of numerous immune-mediators, including chemokines, adenosine triphosphate (ATP), and danger-associated molecular patterns (DAMPs), which leads to activation of microglia, namely microgliosis (Grace et al., 2014). Microglia are phagocytic cells that reside in the brain and spinal cord, constantly monitoring the surrounding tissue for deviations. Neuronally derived mediators trigger the release of several cytokines and other inflammatory mediators, such as brain-derived neurotrophic factor (BDNF) from activated microglia, as well as recruitment of astrocytes and infiltrating T cells. Astrocytes are another form of glial cell that participate in formation of the blood brain barrier, regulate cerebral blood flow, provide structural and trophic support to the neuronal system, and also influence synaptic transmission by modulating the concentration of neurotransmitters and ions in the synaptic cleft (Hutchinson and Terry, 2019). Endothelial cells within the CNS are also responsive to chemokines released during reactive gliosis and CNS injury. Chemokines released by injured neurons, such as CX_3_C-chemokine ligand 1 (CX_3_CL1, also known as fractalkine) promote transendothelial migration of circulating T cells and monocytes into the CNS through their action on endothelial cells (Grace et al., 2011). When activated, these immunocompetent cells act at the synaptic cleft to modulate signal transmission, typically increasing excitatory signals transmission and decreasing inhibitory transmission, resulting in nociceptive hypersensitivity, a feature of central sensitization (Grace et al., 2014). Altered neural pain processing can persist long after the original injury has healed due to ongoing glial and immune cell influence through the release of soluble mediators (Grace et al., 2014; Baral et al., 2019).

The transition from acute pain to chronic pain is not fully understood and there is significant variation in pain phenotype between individuals, owing to the complex multifaceted nature of pain. Dysregulation of the neuroimmune interface has been shown to be involved in pain chronification, particularly after priming of the immunocompetent CNS cells through prior injury or illness (Grace et al., 2014; Moriarty et al., 2019; Adcock, 2021).

## Neuroimmune Interface in Livestock

There has been increasing interest in examining chronic pain and the neuroimmune consequences of injury and disease in livestock production and welfare. While still in its infancy there have been a few studies investigating the cellular, molecular, and genetic influences on the development of pain following husbandry procedures (Sandercock et al., 2019) and disease (Deng et al., 2018; Herzberg et al., 2020a). The fundamental principles of the neuroimmune interface that we are hypothesizing build upon the wealth of knowledge that is currently available to neuroimmune hypothesis in rodents and in humans and will likely have relevance to the livestock sector. The following sections discuss the available literature surrounding neuroimmune interactions in livestock models. Much of the literature focuses on routine husbandry practices or naturally occurring disease commonly encountering on farm. Established rodent neuropathic models, such as spared nerve injury (SNI), are similar to some practices like tail docking, and thus may mimic pathology and may present opportunities to explore and build upon our understanding of neuroimmune contributions to pain in a variety of mammalian species. We think the past literature in humans and rodents provides a foundation on which the livestock research can continue.

### Routine Husbandry Procedures and Chronic Pain

Tail docking is the amputation of the distal portion of the tail and is a common procedure in several livestock industries. It is typically a management practice to reduce disease such as myiasis (flystrike) in sheep and to prevent tail biting in pigs (Sutherland and Tucker, 2011). Amputation of the tail can be performed in several ways: including application of tight rubber rings to induce ischemia and eventual necrosis; incising the tail with a heated iron knife to cauterize the tissue and blood vessels; or surgically, by using a sharp knife or scalpel blade to cut off the tail. The use of anesthesia and analgesia varies significantly between countries and individual producers. Commonly, this practice is performed in young animals without anesthesia or analgesia due to financial and practical implications as well as the absence of evidence showing significantly improved production outcomes when analgesia is used (Sutherland and Tucker, 2011). Tail docking is acutely painful (Molony et al., 1993; Lomax et al., 2010; Sutherland and Tucker, 2011; Herskin and Di Giminiani, 2018) and there is growing evidence of chronic pain associated with the procedure (Kent et al., 1999; Di Giminiani et al., 2017b; Troncoso et al., 2018; Larrondo et al., 2019).

The legislation directing the age at which husbandry procedures can be performed without analgesia or anesthesia varies between countries (Sutherland and Tucker, 2011). Taking tail docking in lambs as an example; the United Kingdom Mutilations (Permitted Procedures) Regulation (United Kingdom Government, 2007) states that tail docking using a rubber ring may be performed without analgesia or anesthesia only in lambs less than 8 days of age, whereas the South Australia Animal Welfare Regulation (South Australian Government, 2012) states that lambs may be tail docked without analgesia up to six months of age. Australian animal welfare guidelines recommend the use of pain relief when practical and cost-effective methods become available and suggest seeking veterinary advice but do not enforce the use of analgesia until the age of six months (Lloyd and Playford, 2013; AHA, 2014; AWI, 2020). In Australia most sheep farming is extensive, so lambs are gathered and tail docked together at 4–8 weeks of age to avoid repeated handling and herding of animals and increase efficiency. Alternatively, sheep in the United Kingdom are often kept indoors for lambing or kept in smaller flocks and farmers most commonly tail dock sheep using a rubber ring within the first week of life (Sutherland and Tucker, 2011).

Amputation of a limb involves severing of the nerves and can lead to the formation of traumatic neuromas, which can be a significant source of pain in humans. Neuromas are benign proliferations of epineural, perineurial, and endoneurial connective tissue and axons that can form at the end of severed nerves and have been associated with chronic neuropathic pain and conditions such as phantom limb pain (Flor et al., 2006). Neuroma formation following tail docking in livestock has been documented in sheep (French and Morgan, 1992; Larrondo et al., 2019), pigs (Sandercock et al., 2016; Kells et al., 2017), and in dairy cows (Eicher et al., 2006; Troncoso et al., 2018). Castration site neuromas have also been identified in horses and may be related to chronic hind limb lameness and back pain (Bengtsdotter et al., 2019).

Sandercock et al. (2019) demonstrated significant changes in lumbar DRG gene expression aligned with those previously observed in inflammatory and neuropathic pain following tail amputation in piglets at 3- and 63-days of age compared to sham treated piglets. These changes were noted at 1-, 8-, and 16-weeks following docking, with the peak changes occurring at 8 weeks. This provides evidence of the development of chronic pain, as defined by IASP, in piglets following tail docking. Overall, there were more differentially expressed genes in the 63-day old piglets compared to the 3-day old piglets, despite multimodal anesthesia and analgesia only being used in the 63-day old piglets. This may be due to delayed development of neuropathic pain in neonates reported in several species (McKelvey et al., 2015; Fitzgerald and McKelvey, 2016; Adcock and Tucker, 2020b).

Castration, commonly practiced in livestock for population management and safety, may have interesting neuroimmune consequences on the development of persistent pain states. The act of castration itself is known to be intensely painful (Kent et al., 1998; Dinniss et al., 2000; Jongman et al., 2000; Thornton and Waterman-Pearson, 2002; Becker et al., 2012; Masłowska et al., 2020) and it is often done in conjunction with other painful procedures, such as tail docking, branding, mulesing and ear tagging. It has been shown in rodent models that neural development and pain processing differs between sexes (Gregus et al., 2021). In males, microglia appear to have a proinflammatory phenotype, whereas female microglia have an anti-inflammatory phenotype (Villa et al., 2018). This is balanced by the proinflammatory nature of estrogen and the anti-inflammatory nature of testosterone. So, what happens when we remove testosterone at the same time as eliciting multiple painful stimuli, stress, temporary maternal separation, exposure to pathogens through open wounds and increased stocking density in stock yards? This is an area that requires further research and is limited by the relatively short life span of castrated males in the production system, often only living 6-18 months (Hutchinson and Terry, 2019; Small et al., 2021).

### Neuropathic Pain in Neonates

In humans and rodents, it has been shown that neuronal injury as a neonate leads to increased pain sensitivity in later life by priming the spinal neuroimmune response (Taddio et al., 1997; Beggs et al., 2012; McKelvey et al., 2015; Walker et al., 2016; Moriarty et al., 2019; Liu et al., 2020). There is also growing evidence that this is true for precocial species such as lambs (McCracken et al., 2010) and calves (Adcock and Tucker, 2018, 2020b). The implications of these findings in the context of the animal production sphere are significant, suggesting that a large proportion of livestock that have undergone various husbandry procedures at an early age may have increased pain sensitivity as adults. This could negatively affect their natural resilience and ability to respond to injury and disease (Clark et al., 2014; Moriarty et al., 2019; Adcock and Tucker, 2020b; Adcock, 2021). One study found that ewes tail docked at 3–4 days of age displayed significantly more pain-related behaviors during parturition at 24 months of age compared to undocked controls (Clark et al., 2014). These findings suggest a link between the tissue injury and pain of tail docking and later sensitization, potentially *via* neuroimmune mechanisms (Beggs et al., 2012), resulting in more painful parturition (Clark et al., 2014). Long and painful parturition is associated with impaired maternal behaviors in the ewe (Dwyer et al., 2003), consequently increasing the risk of perinatal morbidity and mortality in lambs, leading to substantial financial losses and compromised animal welfare (Jacobson et al., 2020).

The mechanisms for delayed neuropathic pain in neonates are not fully understood, but there is growing evidence from rodent studies that neuroimmune modulation suppresses the release of proinflammatory immune mediators, T-cell infiltration, and microglial activation in the dorsal horn of the spinal cord that would normally result in mechanical hypersensitivity seen with adult neuropathic pain (McKelvey et al., 2015; Fitzgerald and McKelvey, 2016). In a spared nerve injury (SNI) model in neonatal mice there was a significant increase in anti-inflammatory cytokines, IL-10, IL-4, and GATA3 (a transcription factor involved in the production of anti-inflammatory cytokines) and no behavioral indicators of neuropathic pain 7 days after surgery (at postnatal day 10). At 28 days after SNI, when these mice developed mechanical hypersensitivity, there was a shift to a proinflammatory profile in the ipsilateral dorsal horn, with a marked increase in proinflammatory markers including IL-1α, IFN-γ, and TNF-α and evidence of microglial activation (increase in Iba-1) (McKelvey et al., 2015). In adults that have been injured as neonates, the spinal neuroimmune response is primed and thus results in greater microglial reactivity and nociceptive sensitivity to subsequent injury (Beggs et al., 2012; Fitzgerald and McKelvey, 2016; Moriarty et al., 2019).

### Chronic Disease and Pain

Disease associated states of chronic pain are a concerning welfare issue in farmed animals, such as dairy cattle. Lameness causes chronic inflammatory pain that severely affects welfare and reduces production in livestock (Ley et al., 1995; Whay and Shearer, 2017; Herzberg et al., 2020b; Steagall et al., 2021). Repeated or sustained trauma and wear of the hoof leads to inflammation involving the release of cytokines by immune cells. Injured tissue allows the introduction of infectious organisms, further exacerbating inflammation and tissue injury. Proinflammatory mediators decrease nociceptor threshold, resulting in local hyperalgesia (primary hyperalgesia). Increasing chronicity and severity of the tissue injury, and ongoing release of inflammatory mediators, leads to further sensitization of primary afferent nociceptors and progression to central sensitization. This can be demonstrated by the development of secondary hyperalgesia, that is, reduced nociceptive threshold in a dermatome distant to the site of injury. Cows with moderate to severe lameness have been shown to have secondary mechanical hyperalgesia that was still present 28 days after diagnosis despite treatment of the underlying disease and no clinical lameness (Whay et al., 1998). Similarly, severely lame sheep suffering from footrot had persistently reduced nociceptive thresholds even 3 months after initial testing when lameness had resolved (Ley et al., 1995).

Neuroimmune contributions to chronic pain associated with lameness have recently been confirmed in sheep, goats (Deng et al., 2018), and cattle (Herzberg et al., 2020a,b). The lumbar spinal cord dorsal horn in chronically lame dairy cows had significantly higher concentrations of several cytokines (IL-1α, IL-13, IFN-γ, IFN-α, CXCL10, CXCL9, and TNF-α) compared to non-lame counterparts (Herzberg et al., 2020b). Proinflammatory cytokines in the CNS are known to contribute to central sensitization, partly through activation of glial cells and modulation of neuronal functions (Grace et al., 2014; Herzberg et al., 2020b). Proteomic analysis of the dorsal horn of the spinal cord of lame cows revealed expression of heat shock proteins (Hsp), Hsp70 and Hsp 90, which act as endogenous DAMPs in the CNS and have been associated with allodynia in rodent pain models (Grace et al., 2014). These Hsp’s, released from damaged neurons, have been shown to activate spinal glial cells and infiltrating immune cells through the TLR2 and TLR4 pathways, resulting in increased expression of cytokines such as TNF and BDNF, contributing to ongoing immune signaling and propagation of central sensitization (Grace et al., 2014). Increased concentrations of chaperone proteins associated with endoplasmic reticulum (ER) stress were also identified in lame cows and there was particularly strong upregulation of Grp78, a known marker of ER stress that is involved in activation of microglia in rodent models of neuropathic pain (Herzberg et al., 2020a).

### What’s on the Horizon?

Major advances in our understanding of the neurobiology of persistent pain have led to the development of pain biomarker tests in rodents and humans (Grace et al., 2010, 2012; Kwok et al., 2013). Application of these tests to livestock and other animals present exciting opportunities to objectively measure persistent pain from a blood sample. Peripheral immune cells circulate through the brain and spinal cord following peripheral nerve injury. These cells interact with microglia and facilitate persistent pain states (Grace et al., 2011). Toll-like receptors (TLRs) are specialized immune receptors that are important for detecting endogenous and exogenous danger signals during the innate immune response. Toll-like receptors are critical to central immune signaling and play a major role in the activation of glia. Consequently, these receptors have been implicated in persistent pain. TLRs are found in all immune cells that are capable of innate immune detection and the responsiveness of peripheral blood immune cells exposed to TLR agonists *ex vivo* mirrors that of central immune cells (Kwok et al., 2012). Given this finding, a blood sample can be used to identify subjects with enhanced glial reactivity and chronic pain. This finding has been used successfully to separate chronic pain sufferers and pain-free controls in rodents and humans (Kwok et al., 2012, 2013). The innate immune state in livestock still needs to be confirmed. Further investigation is expected to confirm similar immune conditions as rodents and humans, allowing translation of the above tests to livestock with some modifications, as required.

Comparison of mRNA transcriptomes of blood and spinal dorsal horn samples from a rodent model of graded neuropathic pain revealed a significant correlation between expression of blood CX_3_CL1 (fractalkine) and spinal CX_3_CR1 (cognate receptor to CX_3_CL1) and mechanical pain sensitivity (Grace et al., 2012). Fractalkine is a chemokine involved in neuron-to-glial signaling and its receptor is expressed by activated microglia in the dorsal horn of the spinal cord. This ligand/receptor cognate is known to play a mechanistic role in pre-clinical models of neuropathic pain. Thus, fractalkine may be a useful blood biomarker of pain and may indicate pain severity (Grace et al., 2012).

Livestock may be promising translational models of naturally occurring painful conditions in humans (Herzberg and Bustamante, 2021). Sheep are already proving to be useful models of human disease due to their larger size compared to typical rodent models and similar anatomy and histomorphology of regions of interest to human disease, such as the stifle (Chakrabarti et al., 2021) and peripheral nerves (Alvites et al., 2021). Chakrabarti et al. (2021) surgically created osteochondral defects on the femoral condyle of the stifle in sheep and demonstrated increased excitability of the ipsilateral dorsal root ganglions consistent with known neural correlates of pain. Despite this neurological evidence of peripheral hypersensitivity, the sheep showed no evidence of clinical lameness at the time of slaughter 2-6 weeks after the lesion was created. This suggests that early osteochondral defects which can progress to painful osteoarthritis in humans (Chakrabarti et al., 2021) may be missed in sheep. Further investigation of this is warranted to target early treatment and prevention of osteoarthritis in sheep and benefit human patients as well.

## Summary

There is evidence for the existence of chronic pain in livestock, even though behavioral measures do not always accurately reflect the presence or severity of pain. The interaction between the immune system and the nervous system contributes to the development and maintenance of chronic pain. This phenomenon has been demonstrated in a number of species and presents exciting opportunities to develop objective measures of pain, new animal models for further study of chronic pain in humans (Burma et al., 2017; Herzberg and Bustamante, 2021), and new targets for pharmacological management of chronic pain (Hutchinson and Terry, 2019; Grace et al., 2021).

## Author Contributions

CJ: writing and editing. MH, AW, and SF: writing, review, and editing. All authors contributed to the article and approved the submitted version.

## Conflict of Interest

The authors declare that the research was conducted in the absence of any commercial or financial relationships that could be construed as a potential conflict of interest.

## Publisher’s Note

All claims expressed in this article are solely those of the authors and do not necessarily represent those of their affiliated organizations, or those of the publisher, the editors and the reviewers. Any product that may be evaluated in this article, or claim that may be made by its manufacturer, is not guaranteed or endorsed by the publisher.
